# A propos d’une lésion lytique de la 1^ère^ phalange: pensez à la sarcoïdose

**DOI:** 10.11604/pamj.2017.26.210.12076

**Published:** 2017-04-19

**Authors:** Zeineb Alaya, Anis Mzabi

**Affiliations:** 1 Service de Rhumatologie, Hôpital Farhat Hached, Sousse, Tunisie; 2 Service de Médecine Interne, Hôpital Sahloul, Sousse, Tunisie

**Keywords:** Dactylite, sarcoïdose, phalange, radiographie, biopsie osseuse, Dactylitis, sarcoidosis, phalanx, X-ray, bone biopsy

## Image en médecine

La dactylite sarcoïdosique est rare. Nous rapportons l'observation d'une patiente de 65 ans ayant consulté pour une tuméfaction indolore du 4^ème^ doigt de la main gauche évoluant depuis 6 ans. La radiographie de la main (A) a montré une ostéolyse de la 1^ère^ phalange du 4^ème^ doigt avec rupture corticale sans réaction périostée. La radiographie du thorax (B) a révélé des adénopathies hilaires bilatérales avec un infiltrat interstitiel. Le bilan tuberculeux était négatif. La biopsie osseuse a montré à l'étude anatomopathologique des granulomes épithélioïdes et giganto-cellulaires sans nécrose caséeuse. Le diagnostic de dactylite sarcoïdosique était retenu. Les principaux diagnostics différentiels regroupent les étiologies infectieuses surtout la dactylite tuberculeuse, le chondrome, la dysplasie fibreuse de l'os et le kyste osseux essentiel, la goutte et l'hyperparathyroïdie primitive.

**Figure 1:**
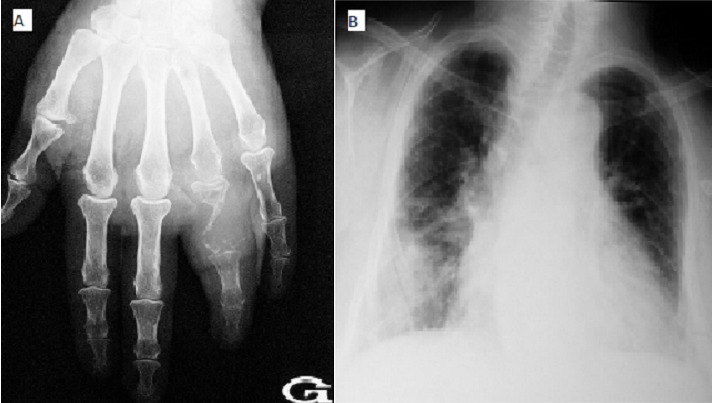
(A) radiographie de la main montrant une ostéolyse de la 1^ère^ phalange du 4^ème^ doigt avec rupture corticale sans réaction périostée; (B) radiographie du thorax révélant des adénopathies hilaires bilatérales avec un infiltrat interstitiel

